# A prospective longitudinal cohort study on risk factors for COVID-19 vaccination failure (RisCoin): methods, procedures and characterization of the cohort

**DOI:** 10.1007/s10238-023-01170-6

**Published:** 2023-09-02

**Authors:** Sibylle Koletzko, Thu Giang Le Thi, Ana Zhelyazkova, Andreas Osterman, Sven P. Wichert, Simone Breiteneicher, Leandra Koletzko, Tobias Schwerd, Stefanie Völk, Tarek Jebrini, Jeannie Horak, Marina Tuschen, Alexander Choukér, Veit Hornung, Oliver T. Keppler, Berthold Koletzko, Helga P. Török, Kristina Adorjan, Members of Riscoin Study Group, O. Keppler, A. Osterman, I. Badell Garcia, M. Huber, P. R. Wratil, K. Adorjan, A. Gryaznova, T. Jebrini, P. Kohl, S. De Jonge, K. Neumeier, S. Koletzko, B. Koletzko, S. Kim-Helmuth, Y. Hao, J. Horak, T. G. Le Thi, B. Puzek, T. Schwerd, H. P. Török, L. Koletzko, S. Breiteneicher, K. Csollarova, A. Choukér, M. Tuschen, K. Biere, T. Wöhrle, S. Matzel, M. Hörl, M. Moser, V. Hornung, J. Rech, C. Ludwig, L. Hansbauer, A. Zhelyazkova, M. Klein, S. Völk, S. Kim-Helmuth, B. Puzek, G. Kastenmüller

**Affiliations:** 1 Department of Pediatrics, Dr. von Hauner Children’s Hospital, LMU University Hospital, LMU Munich, Lindwurmstraße 4, 80337 Munich, Germany; 2 Department of Pediatrics, Gastroenterology and Nutrition, School of Medicine Collegium, Medicum University of Warmia and Mazury, Olsztyn, Poland; 3 Institut für Notfallmedizin und Medizinmanagement (INM), Klinikum der Universität München, LMU München, Munich, Germany; 4 Max von Pettenkofer Institute and Gene Center, Virology, National Reference Center for Retroviruses, LMU Munich, Munich, Germany; 5 German Center for Infection Research (DZIF), Partner Site, Munich, Germany; 6 Department of Psychiatry and Psychotherapy, LMU University Hospital, LMU Munich, Nussbaumstraße 7, 80336 Munich, Germany; 7 Department of Medicine II, LMU University Hospital, LMU Munich, Munich, Germany; 8 Department of Neurology, LMU University Hospital, LMU Munich, Munich, Germany; 9 Department of Anesthesiology, Laboratory of Translational Research Stress and Immunity, LMU University Hospital, LMU Munich, Munich, Germany; 10 Gene Center and Department of Biochemistry, LMU Munich, Munich, Germany; 11 Institute of Psychiatric Phenomics and Genomics (IPPG), LMU University Hospital, LMU Munich, Munich, Germany; 12 Center for International Health (CIH), LMU Munich, Munich, Germany

**Keywords:** SARS-CoV-2, mRNA vaccine, health care worker, inflammatory bowel disease, psychiatric disorder, mobile application

## Abstract

The primary objective of the RisCoin study was to investigate the interplay of genetic, metabolic, and lifestyle factors as well as stress levels on influencing the humoral immune response after at least two COVID-19 vaccinations, primarily with mRNAs, and the risk of SARS-CoV-2 breakthrough infections during follow-up. Here, we describe the study design, procedures, and study population. RisCoin is a prospective, monocentric, longitudinal, observational cohort study. Between October and December 2021, 4515 participants with at least two COVID-19 vaccinations, primarily BNT162b2 and mRNA-1273, were enrolled at the LMU University Hospital of Munich, thereof > 4000 healthcare workers (HCW), 180 patients with inflammatory bowel disease under immunosuppression, and 119 patients with mental disorders. At enrollment, blood and saliva samples were collected to measure anti-SARS-CoV-2 antibodies, their neutralizing capacity against Omicron-BA.1, stress markers, metabolomics, and genetics. To ensure the confidential handling of sensitive data of study participants, we developed a data protection concept and a mobile application for two-way communication. The application allowed continuous data reporting, including breakthrough infections by the participants, despite irreversible anonymization. Up to 1500 participants attended follow-up visits every two to six months after enrollment. The study gathered comprehensive data and bio-samples of a large representative HCW cohort and two patient groups allowing analyses of complex interactions. Our data protection concept combined with the mobile application proves the feasibility of longitudinal assessment of anonymized participants. Our concept may serve as a blueprint for other studies handling sensitive data on HCW.

**Supplementary Information:**

The online version contains supplementary material available at 10.1007/s10238-023-01170-6.

## Background

Severe acute respiratory syndrome coronavirus (SARS-CoV-2) has caused 670,347,729 confirmed infections and 6,823,832 reported deaths from coronavirus disease 2019 (COVID-19) worldwide (as of January 30, 2023) [Coronavirus Resource Center, Johns Hopkins University] [1]. A pandemic of this magnitude significantly challenges healthcare systems. Not only does it push hospitals to the limits of their capacity for patient care [2], but it also puts healthcare workers (HCW) as well as vulnerable patient populations at serious risk.

Vaccination against COVID-19 is an effective measure to combat the consequences of the pandemic. However, the extent and duration of effective protection following COVID-19 vaccination vary between individuals. The immunological response to COVID-19 vaccines and, thus, the protection achieved may be influenced by several factors, including the type of vaccine (mRNA, vector-based) [3], host factors (e.g., age, genetics, health) [4], and exogenous factors (e.g., immunosuppressive therapy, lifestyle, diet, stress) [5]. Studies of B- and T-cell responses [6] and neutralizing capacity [7] in healthy adults following vaccination with different COVID-19 vaccines or combinations of different vaccines show very high variability [8, 9]. Chronic exposure to stressors leading to the release of stress hormones may be one of the underlying factors that impair an effective immune response and affect the human immune system and its humoral and cellular functions [10]. The individual stress response may also play a role in an inadequate response to COVID-19 vaccination and the risk of breakthrough infections [11].

Lifestyle and physical inactivity may be other modifiable factors that influence immune response and susceptibility to infectious diseases [12]. The impact of nutritional status on the response to various vaccines has been demonstrated in numerous studies. For example, a systematic review and meta-analysis of nine studies involving 2367 participants found decreased serological protection against influenza A virus subtype H3N2 and influenza B virus in the presence of vitamin D deficiency [13]. Further, a randomized controlled intervention study showed better vaccine response to pneumococcal vaccine in older people (65–85 years) who consumed ≥ 5 servings of fruits and vegetables daily compared with ≤ 2 servings [14]. In addition, determining the plasma metabolomic profile may help to understand the interaction of genome, environment, and intermediate processes that influence immune function and vaccine response [15]. The metabolome can help to identify the underlying factors that influence the modulation of the immune response and may elucidate the mechanisms of interaction between psychosocial stress and the immune response [16].

Another important aspect that may provide indicators of vaccine failure is the study population. Controlled pivotal trials of COVID-19 vaccine efficacy do not fully reflect the extent of differential vaccine responses in the general population [17, 18] because they exclude participants above a certain age and those with underlying diseases. Patients with primary or secondary immune dysfunction, such as inflammatory bowel diseases (IBD), rheumatic, allergic, autoimmune diseases, or multiple sclerosis, were excluded from the registration studies of the vaccines available in Germany, such as BNT162b2 (Comirnaty® by BioNTech & Pfizer), mRNA-1273 (Spikevax® by Moderna), ChAdOx1 (Vaxzevria® by AstraZeneca), and Ad25.COV2-S (JCOVDEN® by Janssen-Cilag & Johnson & Johnson). No safety or efficacy data, including the magnitude and duration of the vaccine response compared with the general population, were available at the time the vaccines were licensed. Nevertheless, for other diseases, such as influenza, immunosuppression is known to attenuate the vaccine response [19]. Along these lines, first publications in patients under immunosuppressive therapy have already shown that, depending on the disease and the medication, the immune response to SARS-CoV-2 infection detected by PCR and COVID-19 vaccination may be severely attenuated or even absent [20–22].

To investigate the various aspects of immunity to SARS-CoV-2 infection, the German Federal Ministry of Education and Research (BMBF) established the COVIM research program (EudraCT: 2021-001512-28). However, this program did not include studies on genetics, immune metabolism, effects of diet and associated metabolic status, or psychosocial stress to elucidate the large inter-individual variability in the immune response to infection or the COVID-19 vaccine. Therefore, in collaboration with the COVIM consortium, we aimed to fill this gap with the present study on risk factors of vaccination failure (RisCoin), a longitudinal prospective monocentric observational cohort study. Our study may help to generate hypotheses about whether and to what extent specific genes or polymorphisms, stress, and other lifestyle or metabolic patterns may influence the vaccine response and the risk for breakthrough infections. Strategies to influence modifiable risk factors could be implemented, taking advantage of the high motivation of the population to protect themselves effectively against COVID-19. This may also reduce the risk of new chains of infection and the emergence of SARS-CoV-2 variants. The RisCoin study may provide new insights into the functionality of the immune system, which may help to improve the vaccination response to different vaccines or to develop biomarkers that reflect vaccination success.

In this manuscript, we present the objectives and design of the RisCoin study, the enrollment, and follow-up process, the collection of bio-samples, the implementation of a strict data protection concept, and the characteristics of the study population, including three sub-cohorts: healthcare workers (HCW), IBD patients on immunosuppressive therapies and patients with psychiatric disorders. We used a mobile application (study app) that allowed anonymous two-way communication between participants and study managers, including weekly self-reported information on booster vaccinations, report of post-vaccination clinical symptoms, breakthrough infections, and SARS-CoV-2 symptoms to the data platform, and secure delivery of serological results to participants.

## Study objectives

### Primary objective

The primary objective is to investigate whether genetic, metabolic, or lifestyle factors are associated with the magnitude and expression of the immune response after SARS-CoV-2 immunization, taking into account known factors influencing the immune response of COVID-19 vaccination, such as vaccine type, the interval between first and second or booster vaccination, age and presence of primary or secondary (possibly drug-induced) immunodeficiency.

The primary endpoints to answer the question of immune response and risk of vaccine failure are (1) concentrations of IgG type antibodies against SARS-CoV-2 spike protein and some variants of interest and their neutralizing capacity in blood samples and (2) frequency of breakthrough infections after at least two COVID-19 vaccinations (basic immunization) assessed by questionnaires at enrollment and follow-up visits, measurement of IgG type antibodies against SARS-CoV-2 nucleocapsid protein, in addition to the information reported by the participants via the study app, including results of antigen and PCR tests as well as symptoms.

### Secondary objectives

#### Survey and study app-related objectives:

- Acceptance of HCW to participate in an anonymous online survey and to monitor vaccinations, infections, and symptoms via the mobile application (study app).
- Technical requirements, benefits, and limitations of the study app, which was developed for the RisCoin study.

#### Virological-methodological objectives:

- Neutralizing antibody capacity after primary and booster vaccination against various proteins of different variants of concern in relation to antibodies against viral spike protein.
- Quantification of vaccine antibodies measured as antibody concentrations against the spike protein and dependence of these on parameters collected in the questionnaire and results of other work packages (see above).
- Differentiation of antibody characteristics (i.e., avidity) in purely vaccine-induced immunity versus mixed immunity with preceding or breakthrough infection.

#### Epidemiological objectives

- Symptoms and severity of breakthrough infections in the study population.
- To determine the dynamics of the incidence of SARS-CoV-2 infections in HCW over the course of the pandemic.

#### Psychological objectives

- Stress levels in the three sub-cohorts during the study period.
- Stress levels of hospital employees in different occupational groups and their area of assignment.
- Correlation between stress score assessed by the Perceived Stress Questionnaire (PSQ) and measured stress markers in blood and saliva.

#### Metabolic objectives

- Influence of dietary habits and intake of supplements and vitamins on vaccination response.
- Influence of consumption of noxious substances (cigarettes, alcohol) on vaccination response.

## Methods

### Study design and subject population

RisCoin is a prospective, longitudinal, observational cohort study at the LMU University Hospital in Munich, in cooperation with the Division of Infectious Diseases and Tropical Medicine at LMU University Hospital and the COVIM-Consortium in the framework of the German Network University Medicine (NUM). The study design is depicted in Supplementary information 1.

RisCoin comprises three groups of participants, all of whom were required to be vaccinated against COVID-19 at least twice and ≥ 4 weeks before enrollment:

- 1. Healthcare workers (HCW) including trainees at the LMU University Hospital ≥ 18 years of age;
- 2. Patients with inflammatory bowel disease (IBD), including Crohn's disease, ulcerative colitis (UC), or IBD-unclassified, aged 12 years or older, and under the care of the Pediatric or Adult IBD clinic of the LMU University Hospital. This cohort served as a disease control group with a risk of reduced vaccine response due to immunosuppressive drug therapies;
- 3. Immunologically healthy patients with mental disorders from the Department of Psychiatry of the LMU University Hospital were enrolled as a disease control group with a hypothesized risk of high-stress levels.

Subjects were excluded if they had received a blood transfusion, plasma products, or immunoglobulins in the previous 60 days.

### Enrollment and informed consent

RisCoin study information and informed consent form were available to all HCW on the institutional intranet to review and download. HCW and trainees received the link to the intranet page and start date through the regular electronic information on SARS-CoV-2-related issues provided by the Pandemic Board of the LMU University Hospital. IBD patients received study information through newsletters on SARS-CoV2-related issues in IBD sent electronically and by post since the start of the pandemic [23], as well as during their regular IBD clinic visits. Patients with mental disorders were informed in writing and orally by study team members in the psychiatric wards.

We recruited participants from October 7, 2021, to December 16, 2021 (Supplementary information 2). All hospital employees and some IBD patients were recruited centrally at the two hospital sites (Campus Großhadern and Campus Innenstadt). Most IBD patients and all psychiatric patient groups were recruited in the respective departments or outpatient clinics.

During three weeks in October and two weeks in December 2021, recruitment was combined with the booster vaccination organized by the LMU University Hospital. The LMU University Hospital used the mRNA vaccine BNT162b2 (BioNTech/Pfizer) for the basic immunization (vaccination 1 & 2) and for the booster vaccination offered in October 2021 [24]. During the booster vaccination period in December 2021, BNT162b2 was only offered to participants < 30 years of age and pregnant women regardless of age, while all others received mRNA-1273 (Moderna). Participants were also enrolled if they had received their basic immunization outside of the hospital with other mRNA-based or non-mRNA-based vaccines. Participation in the RisCoin study was entirely voluntary. The booster vaccination was not mandatory for study participation. Vice versa, booster vaccination was regularly offered to HCW also if they decided not to participate in the RisCoin study.

All HCW, psychiatric patients, adult, pediatric IBD patients, and their caregivers received verbal information from study physicians about the study objectives, planned examinations, including genetic testing, data protection, and the two-way communication via study app designed for RisCoin. Participants were informed that they would receive results of the serological tests for antibodies against the S- and N-antigen of SARS-CoV-2 and the neutralizing capacity of their antibodies against SARS-CoV-2 and current variants of concern (e.g., Omicron), but not individual results for genetic, metabolomic, or stress markers. All questions were answered before participants and/or caregivers gave their written informed consent. The informed consent form (ICF) allowed participants to consent that any remaining bio-material could be used in an irreversibly anonymized form for future research projects. In contrast, the remaining DNA bio-material from all participants had to be destroyed after the analysis for RisCoin was completed.

To manage the recruitment of up to 200 HCW per day, each participant passed through four different stations (registration and examinations), each with study members trained for specific tasks (Table 1).

**Table 1:** RisCoin enrollment in four stations

**Station 1**	
• Study physicians informed participants about the study and answered their questions	
• Participants and study physicians signed the informed consent form (ICF),	
• Participants received a copy of the signed ICF; the original ICF was collected in a secured container	
**Station 2**	
• The study team handed participants a study kit labelled with the Kit-ID, including	
• a welcome letter providing their unique Contact-ID, a QR code to access the initial questionnaire, and a QR code to link the study app on their smartphone with the master file prepared for the particular Contact-ID,	
• four vials to collect venous blood samples labelled with the Tube-IDs,	
• a card to collect dry blood spots (DBS) labelled with the DBS-ID and	
• a cotton-swab tube to collect saliva labelled with the Tube-ID	
• The study kit was prepacked and labeled with the individualized Tube-IDs linked with Contact-ID, the initial questionnaire, and the assigned QR code for the study app in the welcome letter (Supplementary file 2)	
• The study team instructed each participant to download, install, and activate the study app. All important functions were explained, especially where the participant can find their individual Contact-ID, measurement results, report weekly their symptoms, and how to send messages to the RisCoin team	
**Station 3**	
• Intravenous blood sampling for SARS-CoV-2 serology, genetics, metabolomics, and stress markers. Saliva sampling with cotton swab	
• If needed, the samples were immediately cooled and transported within hours to the respective laboratories for further processing	
**Station 4**	
• Capillary blood was collected from the fingertip to fill five circles on a Dried Blood Sampling (DBS) card	
• The blood-filled DBS cards were collected in special boxes to be dried for at least 24 h	

### Follow-ups

Two follow-up visits with blood sampling for serological measurement of antibodies against SARS-CoV-2 were offered to all HCW and IBD patients, whom (a) had received a booster vaccination at least four weeks prior to the date of the follow-up visit or (b) had a confirmed (by PCR test) or suspected breakthrough infection with clinical symptoms, positive antigen test or had close contact to an infected person with a positive PCR test. Serological testing was not offered to psychiatric patients since almost all of them were not followed up in the clinic after discharge. All follow-ups were communicated to the participants via the intranet page, the newsletter from the Pandemic Board, and the study app. The first follow-up was performed from December 13, 2021, to March 15, 2022, and the second follow-up from September 19, 2022, to October 6, 2022, one year after enrollment in the RisCoin study (Supplementary information 2).

### Data protection concept and ethical approval

The study was conducted in accordance with the Declaration of Helsinki, the International Conference on Harmonization guidelines for Good Clinical Practice (ICH GCP E6 (R2)) and in compliance with the European General Data Protection Regulation 2016/679 (EU-GDPR). The Ethics Committee of the LMU Munich approved the study protocol on September 21, 2021 (Project Number: 21-0839), with acceptance of amendments on February 22, 2022, and May 4, 2022. The data protection concept was approved by the LMU data protection officer on September 15, 2021 and the amendment for the second follow-up on September 8, 2022.

Since the sponsor of the study, the Board of Directors of the LMU University Hospital, was also the employer of the enrolled HCW, and sensitive and genetic data were collected, we developed a multi-level protection scheme to ensure the security of participants' data (Supplementary information 3). All data and bio-materials were double-pseudonymized during recruitment and follow-up, and irreversibly anonymized six months after recruitment ended.

Prior to the irreversible anonymization, the identity logs (ID logs) with the personal data were stored outside the hospital by an independent Trusted Third Party at the Faculty of Medicine of LMU Munich. Once the electronic ID logs had been transferred to the Trusted Third Party, the participants became anonymous to the RisCoin study team (Supplementary information 3). No RisCoin team member had and has access to the ID logs. On June 30, 2022, the Trusted Third Party irreversibly destroyed the electronic ID-Logs. Consequently, the study participants were irreversibly anonymized from July 1, 2022, onwards.

The RisCoin database does not contain any identifiable personal data. Furthermore, the RisCoin database is not connected to the clinical workplace which contains employee personal data.

We used CentraXX software (KAIROS GmbH, Germany), which had already been approved and used in several research studies and biobanks at the LMU University Hospital. Data in CentraXX are protected by multiple security levels and access rights. The system allows different levels of access for different organizational units, i.e., the RisCoin study team (low level) and the RisCoin administrator (high level) (Supplementary information 3). Unauthorized persons did not and do not have access to RisCoin data.

To reduce the risk of mislabeling and sample mix-ups during recruitment, we prepared 5000 potential participants in the RisCoin database, the maximum number we had targeted. The program assigned a RisCoin-ID, Contact-ID, a Kit-ID for study kit to collect the biological samples, including six Tube-IDs for the whole blood sample vials, a saliva container, and a dried blood spot (DBS) card.

### Initial questionnaire at baseline

The initial questionnaire included:

A. General questions on demographics and occupational situation.
B. Questions on SARS-CoV-2 infection prior to inclusion in RisCoin.
C. Questions on COVID-19 vaccination and immunization.
D. Questions on pre-existing health conditions and allergies.
E. Questions on regular medication, intake of vitamins and supplements, diet, and lifestyle.
F. Questions on stress, psychosocial burden, and resilience (only for adult participants ≥ 18 years old).

To compare our results with those of the COVIM study conducted Germany-wide led by Charité, Berlin, Germany [25], we adapted general questions on demographics (e.g., age, sex, weight, height, educational, occupation, number of persons in the household, pre-existing health conditions, concomitant diseases, tobacco, alcohol consumption, and regular medication use, especially immunosuppressive drugs). We collected additional data on regular use of vitamins and supplements, dietary patterns (e.g., consumption of meat, fish, fruit, vegetables, and exclusion of certain foods, pescatarian or vegan diet). Stress and psychosocial distress were assessed with standardized and validated instruments with the consent of the respective authors, including the German Perceived Stress Questionnaire (PSQ) with 20 questions [26, 27], and three of the six questions from the Brief Resilience Scale (BRS) [28]. To avoid confusion, only BRS questions with the 5-point Likert scale from “strongly disagree” (1 point) to “strongly agree” (5 points) were included in the initial questionnaire [28], while questions with reversed scales but the same meaning were not used.

Participants took approximately 15–20 min to complete the initial questionnaire on the Castor EDC online platform. The study team volunteered to assist international participants with language barriers by translating and clarifying questions. A paper and pen version were offered to patients with mental disorders recruited at the Department of Psychiatry and to participants who were not confident with the online survey or had difficulties accessing the internet. The majority of participants completed their initial questionnaire on the day of enrollment and bio-sampling; most of the remaining participants completed the online survey within a few days of enrollment or after a reminder, at the latest by the end of March 2022.

### Weekly questionnaire on booster vaccinations, breakthrough infection, and SARS-CoV-2 infection-related symptoms

Participants were asked to complete a short weekly questionnaire in the study app on clinical symptoms of a possible SARS-CoV-2 infection, their severity, date and type of a COVID-19 booster vaccination, a breakthrough infection with the date of their PCR test. Monitoring via the study app was possible by using only the contact ID and no other identifiers of the participants.

### Sampling for measurements of antibodies against SARS-CoV-2

Blood samples for serum preparation were collected in S-Monovette® Serum CAT/7.5 ml neutral (SARSTEDT #01.1601, Sarstedt AG & Co, Nümbrecht, Germany) and stored at room temperature for a maximum of 8 h. After courier transport to the laboratory for virological diagnostics at the Max von Pettenkofer Institute of the LMU Munich, blood samples were stored at 4°C and centrifuged within 24 h after venipuncture (3600 rpm for 8 min at room temperature using Hettich Centrifuge Rotanta 460 (Hettich GmbH & Co.KG, Tuttlingen, Germany). Two serum aliquots were stored at 4 °C up to three months until further processing and at -20 °C for longtime storage afterward. The storage process for serological investigations has been optimized to avoid multiple freeze–thaw cycles. For longtime storage, the samples were pipetted into MegaBlock® 96-well plates (Sarstedt AG & Co, Nümbrecht, Germany) using a Beckman Biomek NX^P^ S8 (Beckman Coulter, Inc., Indianapolis, USA). Antibodies against SARS-CoV-2 nucleocapsid protein (anti-N) and against the receptor binding domain (RBD) of the spike protein (anti-S) were determined using Elecsys® Anti-SARS-CoV-2 [29] and Elecsys® Anti-SARS-CoV-2 S [30] (Roche, Basel, Switzerland), respectively, in Cobas e 411 analyzer (Roche, Basel, Switzerland) according to accredited routine laboratory standards and the manufacturer's recommendations. For anti-N, a very high specificity was approved in a previous study [31]. The applied assay generated a semi-quantitative result for anti-N (given as COI (cutoff index)) and a quantitative result for anti-S, which is harmonized with the WHO standard (1 U/mL (Elecsys) = 1 BAU/mL (WHO standard)) by the manufacturer [30]. Samples with anti-S antibody concentrations above the upper limit of quantification were manually diluted with Roche Diluent Universal buffer until an absolute quantitation was achieved. Antibody titers were read and interpreted for plausibility and repeated if necessary. Original S-Monovette® were kept for the duration of the study and were discarded afterward.

### Sampling of saliva and blood samples for measurement of stress markers

#### Saliva

Samples were collected with Salivette® Cortisol (SARSTEDT #51.1534.500, Sarstedt AG & Co, Nümbrecht, Germany) according to manufacturer's instructions. After centrifugation (1000 rpm for 2 min at room temperature), saliva was transferred under sterile conditions into three aliquots and stored at -80 °C until further processing.

#### EDTA blood

Venous blood draws were performed with S-Monovette® EDTA K3E/4.9 ml (SARSTEDT #04.1931.001, Sarstedt AG & Co, Nümbrecht, Germany), and samples were stored on crushed ice immediately. Two aliquots of whole blood were stored at −80 °C until further processing. The remaining EDTA blood samples were centrifuged (2500 rpm for 5 min at room temperature), and EDTA plasma was collected in five aliquots and stored at −80 °C until further processing. The monovettes with residual blood pellets were stored upright at -80 °C.

Measured markers in saliva, EDTA whole blood, or plasma include viral parameters Epstein-Barr virus (EBV), Torque teno virus (TTV) or hormonal and immunological profiles (e.g., including testosterone, cortisol, 2-arachidonoylglycerol, N-arachidonoylethanolamine, secretory IgA). Details regarding processing and data analysis will be given in follow-up reports.

### Sampling for genetic analysis

#### EDTA blood

Venous blood draws were collected with S-Monovette® EDTA K3E/4.9 ml (SARSTEDT #04.1931.001, Sarstedt AG & Co, Nümbrecht, Germany) and kept at room temperature for a maximum of 8 h. On the same day of the enrollment, blood samples were transported and stored at −80°C at the Department of Psychiatry and Psychotherapy, LMU Hospital, or at the Gene Center of LMU Munich until further processing.

EDTA blood samples were processed for automated DNA extraction and genotyping at Life & Brain GmbH (Bonn, Germany). Automated DNA extraction was performed from 200 µl EDTA blood in batches of 96 samples on a PerkinElmer chemagic™ 360 and the chemagic™ DNA Saliva 600 Kit H96 kit. Genotyping was performed on the Illumina Infinium Global Screening Array (GSA) v3.0 + MD using a semi-automated protocol. All laboratory procedures were performed in accordance with the manufacturer's instructions. Illumina raw intensity files (.idat) were uploaded together with the Illumina GSA v3.0 + MD manifest (.bmp), and a corresponding cluster file (.egt) into the GenomeStudio v2.0 software and genotypes was subsequently exported to PLINK format for genome-wide association analysis.

All remaining blood samples for genetic analysis were destroyed after processing for DNA extraction and genotyping.

### Sampling for metabolomic analysis

Blood samples for metabolomic analysis were drawn into S-Monovette® Lithium-Heparin (LH)/1.2 ml (SARSTEDT # 06.1666.001, Sarstedt AG & Co, Nümbrecht, Germany). Samples were stored on crushed ice immediately for a maximum of four hours before transport to the laboratory for further processing. After centrifugation, the LH-plasma samples were pipetted into 1 mL Thermo-Matrix tubes in 96-tube racks, scanned via their QR code, and stored at -80°C before further processing. Note that all Thermo-Matrix tubes as well as the 96-tube racks had individual barcodes, human-readable codes and QR codes.

#### Sample preparation

50 µL of LH-plasma sample is added to 450 µL of methanol, which contains a mixture of isotopically labeled internal standards for two metabolomics platforms (amino acids and organic acids of the TCA cycle) and two lipidomics platforms (phosphorylated lipids and acyl-carnitines). Sample preparation and LC–MS analysis of amino acids were performed as described by Newton-Tanzer et al. [32]. Ion-pair reversed-phase (RP) liquid chromatography-mass spectrometry (LC–MS/MS) and the organic acids and keto acids of the TCA cycle were performed as described by Lindsay et al. [33] using RP-LC–MS/MS. For phospholipid analysis such as phosphatidyl choline (PC), lyso-phosphatidyl choline (LPC), and sphingomyelin (SM), we used a flow-injection-analysis LC–MS/MS method as described by Rauschert et al. [34].

Carnitine and acyl-carnitines were analyzed with an in-house method based on the LC–MS/MS method by Giesbertz et al. [35] and are described by Marques et al. [36].

#### Instrumentation

Amino acid analysis was performed on an Agilent 1100 system comprised of a binary pump, an auto sampler, and column oven from Agilent Technologies (Waldbronn, Germany) coupled to an API 2000 triple quadrupole mass spectrometer with electrospray ionization (ESI). Acyl-carnitines, organic acids of the TCA cycle, and phosphorylated lipids were analyzed on an Agilent 1200 system comprised of an Agilent 1200 binary pump, an Agilent 1260 multi-sampler from Agilent Technologies (Waldbronn, Germany) as well as a MayLab column oven with 6-column switching valve from MayLab Analytical Instruments Inc. (Vienna, Austria) coupled to an ESI-QTRAP 4000 MS with an ESI Turbo V ion source.

#### Data processing

Data analysis was performed with Analyst 1.6.3 and metabolite quantification with MultiQuant 3.0 from Sciex (Darmstadt, Germany). Quantitative FIA results were generated with an in-house R-script for isotope correction, background subtraction, and lipid quantification. For accurate statistical data cleaning, normalization, and processing, six quality control samples (QC; pooled sample plasma) per 96-micro well plate (analysis batch) were co-analyzed with the samples. For LC–MS/MS system performance check, two commercial control plasmas ClinCheck®, CP-I and CP-II from Recipe (Munich, Germany), were co-analyzed in duplicates per analysis batch.

## Data management

RisCoin data were reviewed for plausibility, correctness, consistency, data type, range and errors, and outliers were detected according to the standard data cleaning framework to ensure data integrity and enhance data quality [37]. Cross-checks of data collected at enrollment via the initial questionnaire, the short questionnaire at follow-up, and the weekly questionnaire retrieved from the study app were performed continuously after each follow-up period, particularly on the date and type of COVID-19 vaccination and the date of SARS-CoV-2 infection confirmed by PCR testing. The use of the study app made it possible to communicate with participants without their personal data to obtain their confirmation of correct information. Inconsistent or implausible responses from participants in the text descriptions of the initial questionnaire on specific items such as regular medication use, immunosuppressive drugs, and type of allergy were continually reviewed and, if necessary, corrected and validated by medical experts and scientific researchers.

### Statistical analysis

Data analysis was performed using SAS 9.4 (Statistical Analysis Software, SAS Institute Inc., Cary, NC, USA). Descriptive statistics were presented to describe the characteristics of the study population stratified into three cohorts, including HCW, patients with IBD, and patients with mental diseases.

We report continuous variables as median (inter-quartile range from 25 to 75th quartile, IQR) and categorical variables as frequencies and proportions in percent (%).

The detailed analyses of each work package (e.g., genetics, metabolomics, stress markers and life style factors) and their combined analysis will be described in the respective publications.

## Results

### Characteristics of the participants at baseline

A total of 4415 participants with at least one bio-specimen were recruited, of whom 285 had to be excluded for the following reasons: (a) failure to complete the initial questionnaire (*n* = 268), (b) invalid consent form (*n* = 4), failure to provide age (*n* = 11), or HCW being under 18 years of age at enrollment (*n* = 2). Of the remaining 4130 participants, 15 were excluded due to the lack of a blood sample for anti-SARS-CoV-2 serology. The final cohort included 4115 participants, of whom 3816 were HCW, 180 patients with IBD, and 119 patients with various psychiatric disorders. The basic characteristics of the total cohort and the three sub-cohorts are shown in Table 2, including demographics, information on living conditions at home, employment (full-time or part-time, workplace, patient contact during work), prevalence of different pre-existing diseases in the entire group and the sub-cohorts. Current treatment with immunosuppressive drugs was reported by 1.8% of HCW, 100% of IBD patients, and 3.1% of psychiatric patients. Any type of allergy was reported by 1755 (43%) participants; 30.6% reported allergic rhinoconjunctivitis, 11.9% reported an allergy to at least one drug, 9% to any food, and 6% reported contact allergy to chemicals. Daily or almost daily smoking was reported by 12.0% of all participants but by almost 40% of psychiatric patients. Alcohol consumption was denied by a quarter of participants, 24.2% of HCW, 42.1% of IBD patients, and 37.7% of psychiatric patients (Table 2).

**Table 2:** Characteristics of RisCoin cohorts, *N* = 4115

Factors *n* (%) or median (IQR)	All (*N* = 4115)	HCW (*n* = 3816)	IBD cohort (*n* = 180)	PSY cohort (*n* = 119)
Females	2965 (72.2)	2824 (74.2)	81 (45.0)	60 (50.4)
Age in years, median (IQR) (min–max)	39 (29–52) (12–85)	39 (29–52) (18–73)	42 (30–55) (12–80)	42 (27–54) (19–85)
BMI (kg/m^2^), adults ≥ 18 years, *n* = 4090 median (IQR) (min–max)	23 (21–26) (15–62)	23 (21–26) (15–62)	25 (21–28) (17–54)	25 (23–29) (15–41)
*Healthcare profession*				
Nurses	906 (22.1)	900 (23.7)	5 (2.8)	1 (0.8)
Physicians	680 (16.6)	676 (17.8)	3 (1.7)	1 (0.8)
Administration	752 (18.4)	749 (19.7)	2 (1.1)	1 (0.8)
Others (allied health professionals, service staff)	1181 (28.9)	1168 (30.8)	7 (3.9)	6 (5.1)
Working in laboratories and associated institutes	572 (14.0)	300 (7.9)	163 (90.6)	109 (92.4)
*Employment*				
Full-time employment	2513 (61.1)	2397 (62.9)	83 (46.1)	33 (28.0)
Part-time employment	1230 (29.9)	1177 (30.9)	36 (20.0)	17 (14.4)
Other (e.g., trainee, retired, unemployed)	610 (14.8)	483 (12.7)	62 (34.4)	65 (54.6)
*Working place*				
Primarily at home	308 (7.5)	238 (6.2)	50 (27.8)	20 (17.4)
Primarily in presence	3242 (79.0)	3128 (82.1)	79 (43.9)	35 (30.4)
Equally at home and in presence	362 (8.8)	328 (8.6)	20 (11.1)	14 (12.2)
Does not apply	194 (4.7)	117 (3.1)	31 (17.2)	46 (40.0)
*Participants working in health profession*	3537 (86.1)	3511 (92.1)	17 ( 9.4)	9 (7.6)
*Participants with direct patient contact*, *n* = 3533	2398 (67.9)	2378 (67.8)	14 (7.8)	6 (5.0)
*Main working place, if in direct patient contact*				
Intensive care unit with COVID-19 patients	171 (7.1)	171 (7.2)	0	0
Intensive care unit without COVID-19 patients	254 (10.6)	254 (10.7)	0	0
Standard wards with COVID-19 patients	129 (5.4)	127 (5.3)	2 (14.3)	0
Standard wards without COVID-19 patients	629 (26.2)	626 (26.3)	2 (14.3)	1 (16.7)
Emergency department	81 (3.4)	79 (3.3)	1 (7.1)	1 (16.7)
Outpatient clinic	455 (19.0)	455 (19.1)	0	0
Others (e.g., reception, physiotherapy)	679 (28.3)	666 (28.1)	9 (64.3)	4 (66.6)
*Pre-existing health conditions*				
Cardiovascular disease	319 (7.8)	283 (7.4)	20 (11.2)	16 (14.8)
Chronic pulmonary disease	247 (6.0)	220 (5.8)	15 (8.4)	12 (11.1)
Diabetes mellitus	81 (2.0)	73 (1.9)	5 (2.8)	3 (2.8)
Thyroid dysfunction	632 (15.5)	597 (15.7)	21 (11.8)	14 (13.1)
Hypothyroidism	525 (12.9)	505 (13.3)	13 (7.3)	7 (6.6)
Chronic renal disease	28 (0.7)	19 (0.5)	7 (3.9)	2 (1.9)
Renal insufficiency	7 (0. 2)	3 (0.1)	4 (2.2)	0
Chronic hepatic/gastrointestinal disease	269 (6.6)	82 (2.2)	180 (100)	7 (6.5)
Chronic neurological disease/ disorder	98 (2.4)	83 (2.2)	4 (2.2)	11 (10.4)
Active cancer	12 (0.3)	8 (0.2)	1 (0.6)	3 (2.8)
Cancer in remission	30 (0.7)	25 (0.7)	5 (2.8)	0
Cured cancer	102 (2.5)	93 (2.4)	4 (2.2)	5 (4.7)
Transplantation	8 (0.2)	4 (0.1)	4 (2.2)	0
Chronic hematological disease	26 (0.7)	24 (0.7)	0	2 (2.0)
Rheumatological disease	91 (2.2)	81 (2.1)	8 (4.5)	2 (1.9)
Chronic immune disease	59 (1.4)	42 (1.1)	12 (6.7)	5 (4.7)
*Allergy*	1755 (43.0)	1630 ( 42.9)	82 (46.1)	43 (39.8)
Drug allergy	489 (12.0)	443 (11.7)	32 (18.0)	14 (13.0)
Food allergy	368 (9.0)	344 (9.1)	17 (9.6)	7 (6.5)
Pollen allergy (allergic rhinoconjunctivitis)	1248 (30.6)	1162 (30.6)	59 (33.1)	27 (25.0)
Allergy against wasps, bee poison	97 (2.4)	92 (2.4)	3 (1.7)	2 (1.9)
Contact allergy with chemicals	245 (6.0)	238 (6.3)	6 (3.4)	1 (0.9)
Pseudo allergy	17 (0.4)	17 (0.4)	0	0
Anaphylaxis in the past	171 (4.2)	150 (3.9)	16 (9.0)	5 (4.6)
*Current chemotherapy and/or radiation in the last 3 months against active cancer*, *n* = 12	3	2	1	0
*Current chemotherapy and/or radiation in the last 3 months against cured cancer*, *n* = 102	1	1	0	0
*Medication that can suppress the immune system (e.g., to treat autoimmune disease, inflammatory bowel disease, rheumatic diseases, or cancer)*, *n* = 4077	252 (6.2)	69 (1.8)	180 (100)	3 (3.1)
*Number of persons living permanently in the same household including the participant*				
Only one	1010 (24.7)	927 (24.4)	42 (23.6)	41 (38.7)
2 persons	1564 (38.3)	1472 (38.8)	61 (34.3)	31 (29.2)
≥ 3 persons	1508 (37.0)	1399 (36.8)	75 (42.1)	34 (32.1)
*Smoking status (tobacco products, e-cigarettes, hookah pipe)*				
Current smoker (daily or almost daily)	489 (12.0)	422 (11.1)	25 (14.0)	42 (39.6)
Current smoker (occasionally)	247 (6.1)	236 (6.2)	9 (5.1)	2 (1.9)
*Alcohol consumption*				
Yes	2863 (70.2)	2721 (71.7)	92 (51.7)	50 (47.2)
No	1033 (25.3)	918 (24.2)	75 (42.1)	40 (37.7)
No more (previous alcohol consumption)	118 (2.9)	95 (2.5)	8 (4.5)	15 (14.2)

*BMI* body mass index, *HCW* health care workers, *IBD* inflammatory bowel disease, *IQR* inter-quartile range, *PSY* psychiatric

Prior to enrollment, 6.5% of the total cohort reported a SARS-CoV-2 infection, thereof 86.8% confirmed by PCR testing, with no significant difference between the three sub-cohorts (Table 3). Almost one in two participants had close contact with a person with a confirmed SARS-CoV-2 infection during the study period. The basic immunization, the first two doses, had been administered with BNT162b2 in 94% of the HCW, but only in 83.3% of the patients with IBD, and 64.1% of the patients with psychiatric diseases. At enrollment, 304 (7.4%) participants had already received a booster vaccination. Of the 2075 participants with only two vaccinations at enrollment, 80.4% planned to get a booster, with large differences between groups (HCW, IBD patients, and patients with psychiatric diseases, 81.7%, 88.9%, and 36.6%, respectively) (Table 3). During the first influenza season after the start of the pandemic (winter 2020/21), 51.1% had been vaccinated against influenza (HCW, IBD, and psychiatric patients 51.6%, 60.3%, and 19.8%, respectively).

**Table 3:** SARS-CoV-2 infection and COVID-19 vaccination at study inclusion, *N* = 4115

Factors *n* (%) or median (IQR)	All (*N* = 4115)	HCW (*n* = 3816)	IBD cohort (*n* = 180)	PSY cohort (*n* = 119)
*SARS-CoV-2 infection prior to study inclusion*	264 (6.4)	242 (6.3)	15 (8.3)	7 (5.9)
*PCR confirmed SARS-CoV-2 infection prior to study inclusion*, *n* = 4028	231 (5.7)	214 (5.7)	12 (6.8)	5 (4.9)
*Contact with confirmed SARS-CoV-2 infected person(s) ever*	1852 (45.1)	1785 (46.8)	38 (21.1)	29 (24.8)
*Contact with confirmed SARS-CoV-2 infected persons or COVID-19 cases, n = 1852*				
Colleague(s)	876 (47.3)	852 (47.7)	13 (34.2)	11 (37.9)
Patient(s)	937 (50.6)	929 (52.0)	5 (13.2)	3 (10.3)
In private environment	682 (36.8)	638 (35.7)	25 (65.8)	19 (65.5)
*First COVID-19 vaccination—vaccine, n = 4088*				
BNT162b2 (BioNTech/Pfizer)	3790 (92.7)	3572 (94)	148 (82.7)	70 (64.8)
mRNA-1273 (Moderna)	129 (3.2)	96 (2.5)	12 (6.7)	21 (19.4)
ChAdOx1 (AstraZeneca AB)	150 (3.7)	118 (3.1)	16 (8.9)	16 (14.8)
Others	19 (0.5)	15 (0.4)	3 (1.7)	1 (0.9)
*Second COVID-19 vaccination—vaccine, n = 3984*				
BNT162b2 (BioNTech/Pfizer)	3785 (95)	3553 (95.8)	156 (89.7)	76 (75.3)
mRNA-1273 (Moderna)	149 (3.7)	113 (3)	14 (8.1)	22 (21.8)
ChAdOx1 (AstraZeneca AB)	43 (1.1)	36 (1)	4 (2.3)	3 (3.0)
Others	7 (0.2)	7 (0.2)	0	0
*Vaccine of the first and second COVID-19 vaccination*				
BNT162b2 (BioNTech/Pfizer)	3697 (92.7)	3486 (94.0)	145 (83.3)	66 (64.1)
mRNA-1273 (Moderna)	120 (3.0)	90 (2.4)	11 (6.3)	19 (18.4)
ChAdOx1 (AstraZeneca AB)	42 (1.1)	36 (1.0)	3 (1.7)	3 (2.9)
Mixed vaccines	128 (3.2)	98 (2.6)	15 (8.6)	15 (14.6)
*Third COVID-19 vaccination prior to study inclusion—vaccine, n = 304*				
BNT162b2 (BioNTech/Pfizer)	285 (93.8)	263 (93.9)	15 (88.2)	7 (100)
mRNA-1273 (Moderna)	19 (6.3)	17 (6.1)	2 (11.8)	0
*Willingness for the booster vaccination against COVID-19 (after the 2nd COVID-19 vaccination), n = 2075*				
Yes	1668 (80.4)	1518 (81.7)	120 (88.9)	30 (36.6)
No	32 (1.5)	29 (1.6)	2 (1.5)	1 (1.2)
I am not sure	375 (18.1)	311 (16.7)	13 (9.6)	51 (62.2)
*Influenza vaccination at inclusion, n = 4095*				
Vaccinated against influenza ever	2842 (69.4)	2666 (70.1)	129 (72.1)	47 (42.3)
Vaccinated against influenza during the last flu season (October 2020–May 2021)	2093 (51.1)	1963 (51.6)	108 (60.3)	22 (19.8)

*HCW* health care workers, *IBD* inflammatory bowel disease, *IQR* inter-quartile range, *PSY* psychiatric

### Follow-ups

Between December 13, 2021, and March 15, 2022, a total of 1784 participants donated blood for the first follow-up serological test for antibodies against SARS-CoV-2, including 1694/3816 (44.4%) of HCW and 90/180 (50%) of patients with IBD. At the time of bio-sampling for the first follow-up, 115/1772 (6.5%) reported at least one SARS-CoV-2 infection since enrollment into the RisCoin study (6.5% of HCW and 5.6% of patients with IBD, respectively). A few patients had submitted more than one serum sample for antibody testing for different reasons, particularly due to close contact with confirmed SARS-CoV-2 infected persons. Since enrollment, 1742/1784 (97.6%) had received a third (first booster) and 10/1784 (0.6%) a fourth COVID-19 vaccination. The type of booster vaccine in the different subgroups and the combination of vaccines are shown in Table 4.

**Table 4:** SARS-CoV-2 infection and COVID-19 vaccination at follow-ups

Factors n (%) or median (IQR)	All (*N* = 1784)	HCW (*n* = 1694)	IBD cohort (*n* = 90)
*A) Follow-up 1 from December 13, 2021, to March 15, 2022*			
Females	1341 (75.3)	1301 (76.9)	40 (44.4)
Age in years, median (IQR) (min–max)	44 (33–55) (18–80)	43.5 (32–55) (18–72)	46 (38–55) (19–80)
*Healthcare profession*			
Nurses	385 (21.6)	383 (22.7)	2 (2.2)
Physicians	324 (18.2)	324 (19.2)	0
Administration	364 (20.5)	363 (21.5)	1 (1.1)
Others (e.g., technical assistants, service staff)	507 (28.5)	505 (29.9)	2 (2.2)
Not working in clinical setup	199 (11.2)	114 (6.7)	85 (94.4)
*PCR confirmed SARS-CoV-2 infection*	115 (6.5)	110 (6.5)	5 (5.6)
*PCR confirmed SARS-CoV-2 infection after 3rd vaccination*	83 (4.7)	79 (4.7)	4 (4.4)
*Contact with confirmed SARS-CoV-2 infected person(s)*	560 (42.9)	543 (43.7)	17 (27.9)
At work with colleague(s)	299 (16.8)	294 (17.4)	5 (5.6)
At work with patient(s)	199 (11.2)	196 (11.6)	3 (3.3)
In private environment (at home or at a private event)	257 (14.4)	246 (14.5)	11 (12.2)
*Third COVID-19 vaccination—vaccine, n = 1742*			
Comirnaty (BioNTech/Pfizer)	1167 (67.0)	1122 (67.8)	45 (51.1)
COVID-19 Vaccine (Moderna)	575 (33.0)	532 (32.2)	43 (48.9)
*Fourth COVID-19 vaccination—vaccine, n = 10*			
Comirnaty (BioNTech/Pfizer)	6	6	0
COVID-19 Vaccine (Moderna)	4	3	1

Factors *n* (%) or median (IQR)	All (*N* = 1024)	HCWs (*n* = 957)	IBD cohort (*n* = 67)
*B) Follow-up 2 from September 19, 2022, to October 31, 2022*			
Females	804 (78.7)	774 (81.0)	30 (44.8)
Age in years, median (IQR) (min–max)	47 (37–56) (12–78)	47 (37–56) (19–72)	48 (36–58) (12–78)
*Healthcare profession*			
Nurses	197 (19.3)	196 (20.5)	1 (1.5)
Physicians	157 (15.4)	156 (16.4)	1 (1.5)
Administration	235 (23.0)	234 (24.5)	1 (1.5)
Others (e.g., technical assistants, service staff)	294 (28.8)	292 (30.6)	2 (3.0)
Not working in clinical setup	138 (13.5)	76 (8.0)	62 (92.5)
*Vaccinated against influenza during the flu season (Oct. 2020–May 2021)*, *n* = 1024	648 (63.3)	602 (62.9)	46 (68.7)
*Vaccinated against influenza during the last flu season (Oct. 2021–May 2022),* *n* = 1009	643 (63.7)	604 (63.9)	39 (60.9)
*SARS-CoV-2 infection (Oct. 2021 onwards), n = 1017*			
No	453 (44.5)	419 (44.1)	34 (51.5)
Yes, 1x	534 (52.5)	504 (53.0)	30 (45.5)
Yes, 2x	29 (2.9)	27 (2.8)	2 (3.0)
Yes, 3x	1 (0.1)	1 (0.1)	0
*PCR confirmed SARS-CoV-2 infection after 3rd vaccination*	454 (44.6)	433 (45.5)	21 (31.8)
*Contact with confirmed SARS-CoV-2 infected person(s)*			
At work with colleague(s) or patient(s)	81 (8.0)	77 (8.1)	4 (6.1)
At home with family member(s)	203 (20.0)	192 (20.2)	11 (16.7)
At a private event	124 (12.2)	118 (12.4)	6 (9.1)
*Third COVID-19 vaccination—vaccine, n = 996*			
BNT162b2 (BioNTech/Pfizer)	633 (63.6)	594 (63.7)	39 (60.9)
mRNA-1273 (Moderna)	362 (36.3)	337 (36.2)	25 (39.1)
ChAdOx1 (AstraZeneca AB)	1 (0.1)	1 (0.1)	0
*Fourth COVID-19 vaccination—vaccine, n = 186*			
BNT162b2 (BioNTech/Pfizer)	167 (89.8)	151 (91.0)	16 (80.0)
mRNA-1273 (Moderna)	19 (10.2)	15 (9.0)	4 (20.0)
*Fifth COVID-19 vaccination—vaccine, n = 3*			
BNT162b2 (BioNTech/Pfizer)	3	2	1

*HCW* health care workers, *IBD* inflammatory bowel disease, *IQR* inter-quartile range

**Fig. 1:**
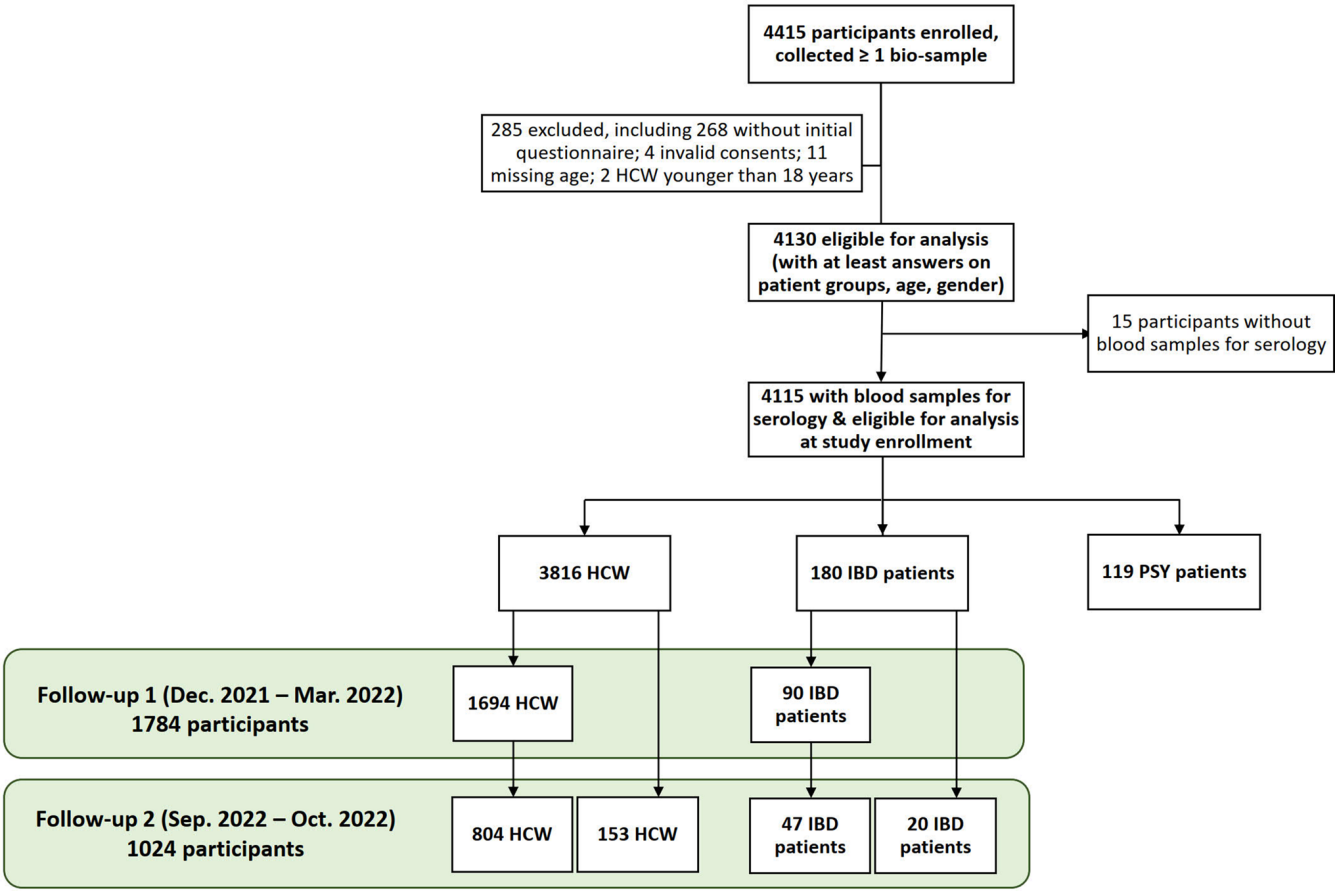
Flowchar of RisCoin cohort with blood samples for serology, *N* = 4115. HCW: Health care workers, IBD: Inflammatory bowel disease, PSY: Psychiatric

Between September 19 and October 6, 2022, 1053 participants donated blood for serological testing, of whom only 181 missed the first follow-up blood sampling (Fig. 1). Since enrollment, more than half of the participants in the second follow-up had experienced at least one SARS-CoV-2 infection.

## Discussion

The RisCoin study investigates biological factors (age, sex, genotype, medical history) and exogenous, largely modifiable factors (lifestyle, diet, stress, use of supplements/drugs, and immunosuppressive therapy) on vaccination response and risk of breakthrough infections over time in > 4000 vaccinated individuals, mostly employees of a large university hospital. Several challenges, both foreseen and unforeseen, arose before and during this ambitious study.

The main challenge in setting up the study was to ensure a secure privacy policy, as the study sponsor was also the employer of the enrolled HCW. It was therefore essential to ensure a strong data protection policy so that HCW could feel confident about providing sensitive data about their medical history (underlying diseases, use of all medications, vaccination status, COVID-19 symptoms, and PCR test results) and consenting to genetic testing. The privacy concept described in the methods section and presented in Supplementary information 3 allowed for bidirectional communication via the study app even after irreversible anonymization of the participants. The concept was presented to and discussed with the members of the ethics committee, the staff council of the LMU University Hospital, and the data protection officer before final approval.

This strict data protection concept certainly contributed to the high number of participating HCW, but it also had drawbacks, as we could only contact participants through general announcements in the hospital and individually through the study app. Consequently, if participants did not answer essential questions in the initial questionnaire (e.g., sub-cohort, age, and sex) and did not respond to reminders and queries via the study app, we had to exclude them from the final analysis. This resulted in the exclusion of 285 participants with unnecessary costs of analyzing their bio-specimens.

Another challenge was the very short timeframe between the award of the grant in June 2021 and the start of enrollment in mid-October 2021. In less than four months, the RisCoin team had to develop a digitized research project, including a data protection concept with study app, an online questionnaire platform, and bio-banking, and to enable a complex logistics system with sufficient personnel for enrollment and bio-sample processing in the various laboratories. Within two months, 4415 participants were enrolled in the study through an extensive recruitment strategy. Enrollment in the study was linked to the vaccination program of the LMU University Hospital [38]. On days when vaccination was offered to HCW, up to 200 participants were recruited. This high caseload and the need to recruit at two sites of our hospitals, 8 km apart, resulted in high staffing requirements at the different sites (Table 1): study physicians for informed consent, nurses for bio-specimen collection, instructors for explaining and activating the study app, and technicians in the laboratories for processing and storing > 20000 bio-specimens. The high daily caseload during enrollment was a potential source of error, but well-prepared logistics resulted in very few participants being excluded due to invalid consent or missing samples. No mislabeling of specimens occurred.

The app-based data collection tool was used for the first time at the LMU University Hospital and had clear benefits but presented a huge challenge. Bidirectional communication was not originally built into the app and had to be developed within two months by our IT team in collaboration with the manufacturer. The provision of a communication solution via app provided participants with a quick and convenient option to contact the study team whenever questions or technical difficulties arose. Participants’ comments, questions, concerns and feedback especially regarding the app’s architecture were taken into consideration in order to improve the user journey throughout the digital individual record. The app provided study participants with information and explanations about their individual serological results, including neutralizing antibodies against Omicron variants. Individual questions from participants could be answered in a timely manner while maintaining participant anonymity, which would not have been possible with an e-mail-based hotline. The team had to respond quickly to the dynamic events of the pandemic and strive to make a relevant contribution to the pandemic response and the safety and health of staff and patients at the LMU University Hospital. Unforeseen problems with the app required ongoing technical support to reduce the number of participants dropping out due to malfunction. Our experience with this tool will be the subject of a separate manuscript.

Overall, there was a high level of willingness to be vaccinated among HCW at the LMU University Hospital [38], which in turn certainly contributed to a large number of participants in the study, including follow-ups. The final cohort of 3816 HCW is representative of the 11000 employees of the LMU University Hospital, associated laboratories, and institutions in terms of age and gender distribution and representation of different workplaces in relation to the risk of acquiring SARS-CoV-2 infection. Two-thirds of the enrolled hospital staff had direct patient contact at work. Allergies were commonly reported in the overall cohort (43%) and in the three subgroups, with higher proportions of contact allergies to chemicals among HCW and higher proportions of drug allergies and previous anaphylactic shock among IBD patients. More female participants with a trend for older age returned for one or both follow-ups, with a similar distribution of workplaces. To allow comparison and potentially data sharing with other consortia assessing the post-vaccine immune response, we harmonized many questions in RisCoin with those provided to the participants of the multicentric COVIM study. COVIM included defined vaccinated patient groups with primary and secondary immunodeficiency from 11 different hospitals all over Germany using 500 HCW as control group.

Finally, the emergence of the Omicron variant in Germany during the second half of the enrollment phase, with the rapid spread and steep increase in SARS-CoV-2 among participants, required a very flexible adaptation by the study team. We started follow-up sampling in December 2021 to provide participants with serological results, including neutralizing antibodies after their most recent vaccination and/or after symptomatic and even asymptomatic infections. In summary, in this manuscript, we have described the study design, data protection concept, and procedures of the RisCoin study. We presented the characteristics of the total cohort and stratified it into three sub-cohorts at enrollment and follow-up. The more detailed analyses and their results of laboratory measurements to answer the primary and secondary objectives of the study will be reported in future publications. If specific genes or polymorphisms or metabolic biomarkers could be identified as the cause of an inadequate immune response in otherwise healthy subjects, these individuals could be prospectively identified in the future and protected through appropriate surveillance and booster vaccinations. Our study is well suited to investigate, e.g., the association of gene variants and immune response correlates after COVID-19 vaccination. Although we did not measure gene expression, we can assess the role of previously identified candidate genes in our genetic association studies. For example, significant eQTL (expression quantitative trait loci) effects have been reported for both PGLYRP4 and HEPHL1 [39]. Should we identify these Single Nucleoid Variants (SNVs) in our genetic association study, we could infer a role for these transcripts in response to COVID-19 vaccination. Such validation analyses are required to confirm identified genetic associations. At the same time, our collected data will also allow us to test such associations in a hypothesis-driven manner. Both approaches are planned in our genetic follow-up study.

If RisCoin identifies modifiable risk factors such as lifestyle factors or stress levels for vaccination failure, strategies could be implemented to take advantage of the population's high motivation to protect themselves effectively against COVID-19. This could also reduce the risk of new chains of infection and the emergence of SARS-CoV-2 mutants. Ideally, identified risk or protective environmental factors may be confirmed in future randomized controlled trials to prove their causality. The RisCoin study may provide new insights into the functioning of the immune system that could help improve vaccine response to different vaccines or develop biomarkers that map vaccination success.

### Supplementary Information

Below is the link to the electronic supplementary material.

Supplementary file 1

Supplementary file 2

Supplementary file3 (DOCX 286 kb)

Supplementary file4 (DOCX 67 kb)

## Abbreviations

Ab: Antibodies
App: Application
BRS: Brief Resilience Scale
COVID-19: Coronavirus Disease 2019
DBA: Database administrator
HCW: Healthcare Workers
IBD: Inflammatory Bowel Disease
ICF: Informed Consent Form
PCR: Polymerase Chain Reaction
PI: Principal Investigator
PSQ: Perceived Stress Questionnaire
SARS-CoV-2: Severe Acute Respiratory Syndrome Coronavirus 2
SNP: Single Nucleotide Polymorphism
