# Supplementary figures and images for: A prospective longitudinal cohort study on risk factors for COVID-19 vaccination failure (RisCoin): methods, procedures and characterization of the cohort
Source: Clin Exp Med. 2023 Sep 2;23(8):4901–17. doi: 10.1007/s10238-023-01170-6 (PMC10725370; doi:10.1007/s10238-023-01170-6)

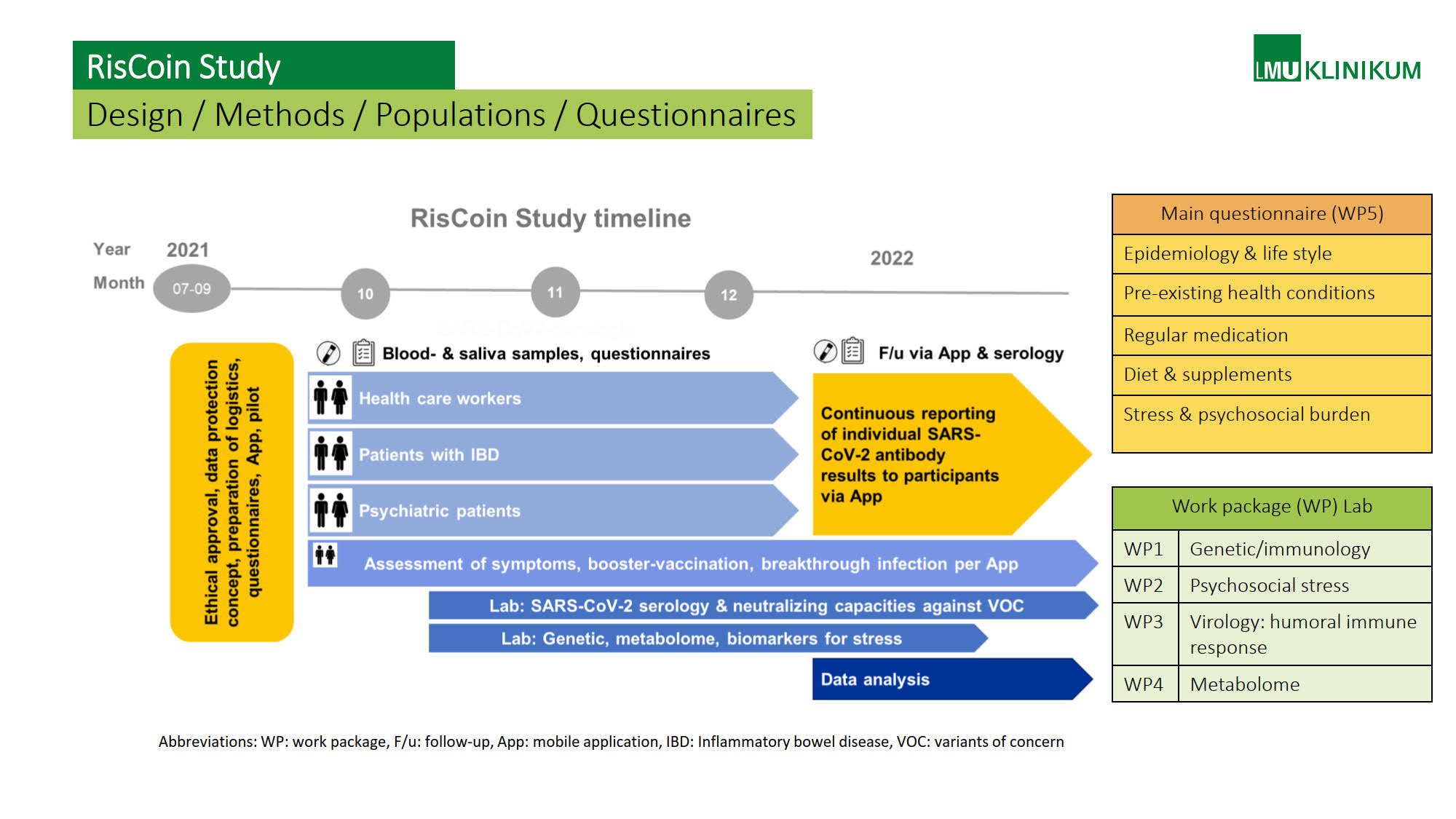

Supplement: Supplementary file 1 [file 10238_2023_1170_MOESM1_ESM.jpg]

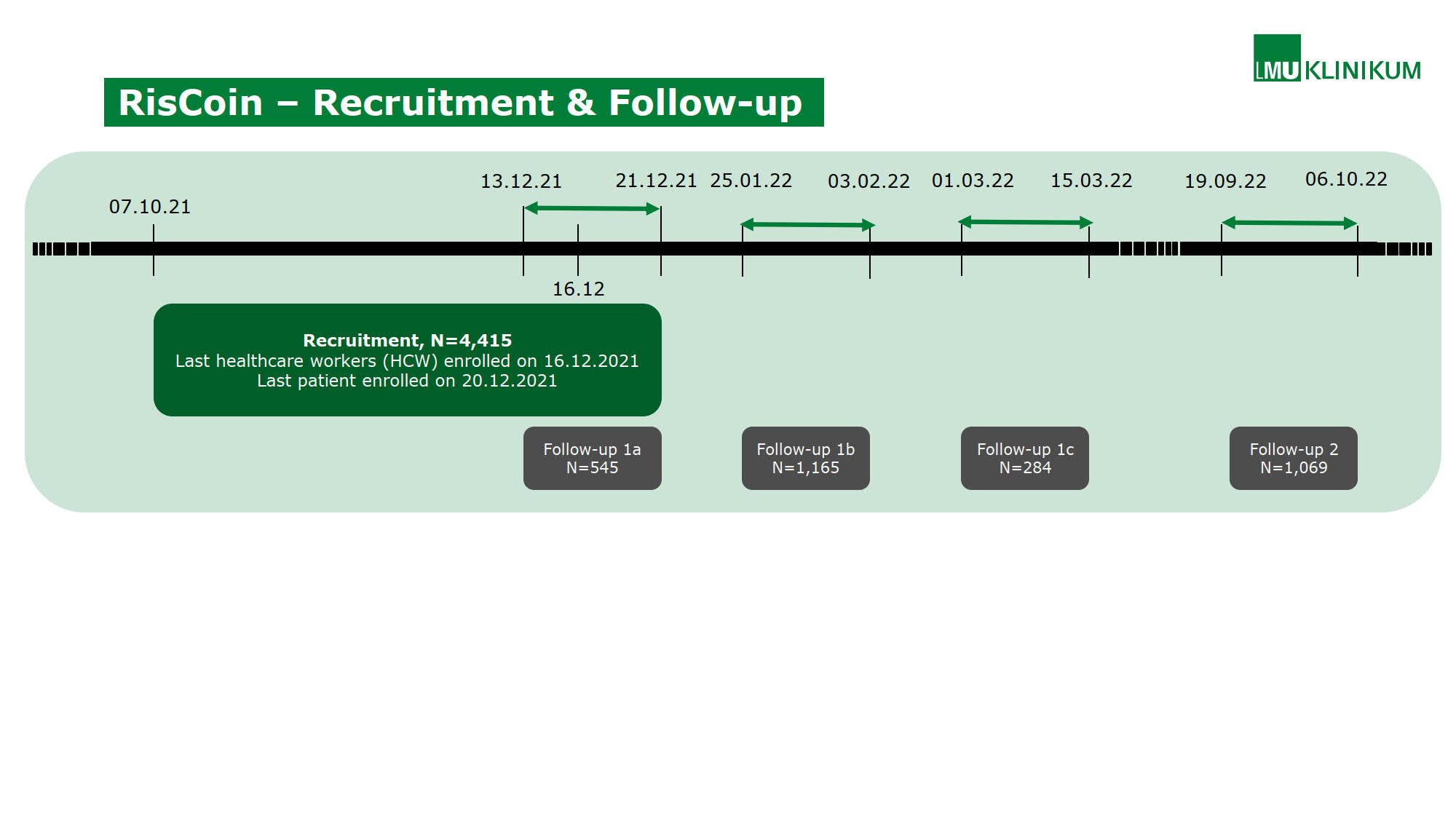

Supplement: Supplementary file 2 [file 10238_2023_1170_MOESM2_ESM.jpg]
