# Supplementary material for: A prospective longitudinal cohort study on risk factors for COVID-19 vaccination failure (RisCoin): methods, procedures and characterization of the cohort
Source: Clin Exp Med. 2023 Sep 2;23(8):4901–17. doi: 10.1007/s10238-023-01170-6 (PMC10725370; doi:10.1007/s10238-023-01170-6)
Supplement: Supplementary file 3 — Supplementary file3 (DOCX 286 kb) [file 10238_2023_1170_MOESM3_ESM.docx]

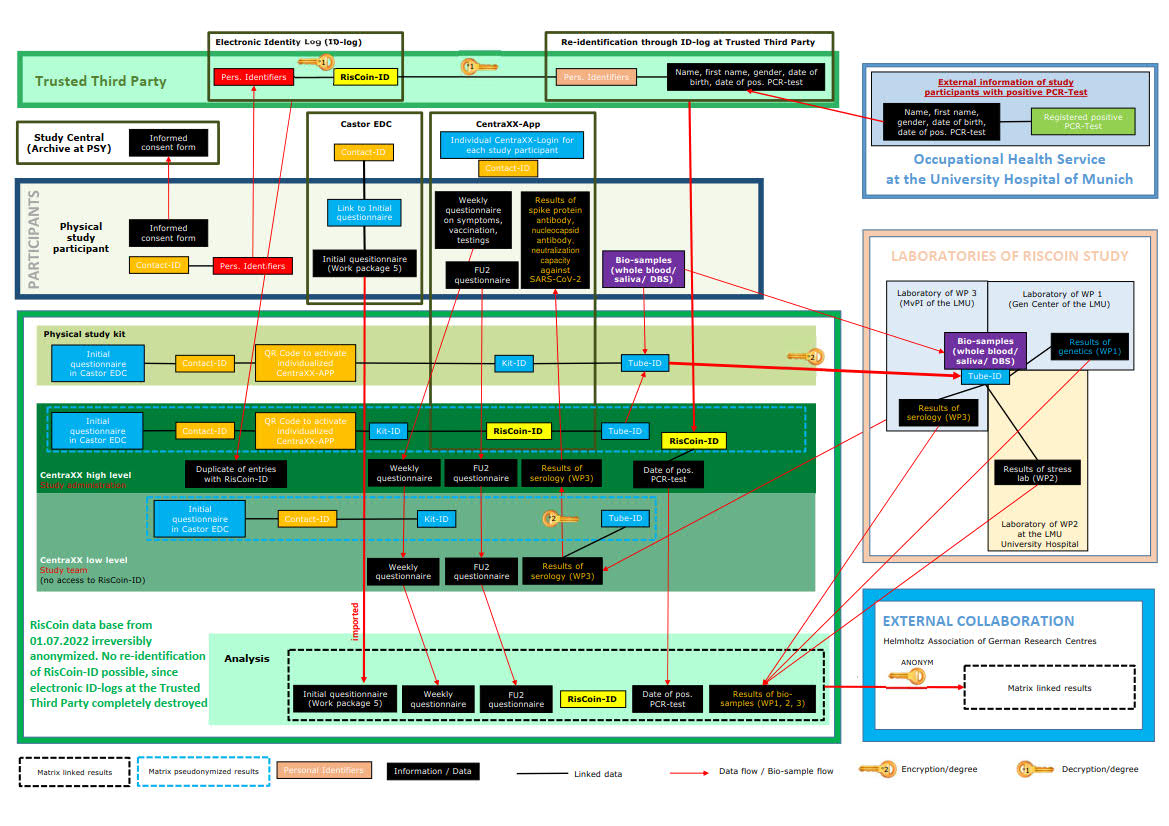
Supplementary information 3: Explanation of the data flow diagram

The RisCoin data security concept used different connected codes for each participant generated prior to inclusion in CentraXX for each participant.

- **RisCoin-ID** is a code allocated to each participant by the CentraXX software at recruitment and consists of study name and a serial consecutive five-digit code. The participants and the study team members have no access to the RisCoin-ID.
- **Contact-ID** is a random six-digit number generated by the CentraXX software. All bidirectional communication from the study personnel to the participants (e.g., to inform about next serological controls or communicate the individual antibody results) and from the participants to the study steam (e.g., to ask questions or to report booster vaccinations or breakthrough infections) go through this Contact-ID via the study app. This is the only ID the participant knows. The study team and the data manager cannot link the Contact-ID with any personal data. The data manager can connect the Contact-ID with the RisCoin-ID and the IDs used for bio samples.
- **Kit-ID** was a random number generated by the CentraXX software, not allowing any identification with the RisCoin-ID, but linked with Contact-ID, the initial questionnaire, all materials in one sample kit, and their assigned QR code for the study app.
- **Tube-ID** was linked to the Kit-ID and had an additional code assigned to each processing laboratory, respectively. The vials labeled with Tube-ID for each blood sample (serology, genetics, metabolome, stress markers), saliva sample, and a card to collect DBS were prepacked in a plastic bag and labeled with the corresponding Kit-ID. The analyzing labs analyzed the bio sample based on the Tube-ID only, with no knowledge of Contact-ID or RisCoin-ID). The lab results were reported into the RisCoin database, which automatically allocated the results based on the Tube- ID to the participant with the RisCoin-ID.
- **QR Code to access an individualized questionnaire** via Castor EDC.
- **QR Code to activate the individualized study app**

Participants' identifiers (name, date of birth, address, and e-mail address) were filled out by the participants on the Informed Consent Form (ICF), which did not include any of the IDs. The original ICFs without IDs were separated from the ID log and kept by the Principal Investigator (PI) in the study archives at the Psychiatric Hospital. The first page of the ICF with the personal information was copied by the study team. Prepared stickers with the Contact-ID and the Kit-ID, provided in the study kit, were attached to this copied page to assign a Contact-ID to the participant. This sheet generated the ID-Log by transferring personal data from the ICF to the electronic ID-Log. Independent personnel not involved in the recruitment process entered the participant details visible on the copied ICF and their assigned Kit-ID into the electronic ID-Log, including participant's surname, first name, date of birth, gender, address, and the assigned Kit-ID. All copied ICFs were destroyed upon completion of the ID-Log. The data manager translated the Kit-ID into the RisCoin-ID and removed the Kit-ID from the electronic ID-Log so that the ID-Log contained only the following information for each participant: RisCoin-ID, surname, first name, date of birth, gender, and address of the participant.In RisCoin Study, only the Trusted Third Party, Institute for Medical Information Processing, Biometry, and Epidemiology (IBE) at the Faculty of Medicine at the LMU Munich, Marchioninistr. 15, 81377 Munich, could link the personal data with the RisCoin-ID. Initially, there were two possible situations for re-identification:

1. Communication of sequencing results of the infecting SARS-CoV-2 variant in the case of breakthrough infection: with the consent of the study participant, the Trusted Third Party received from the Occupational Health Service of the LMU Hospital the surname, first name, gender, and address of the affected person, matched with the corresponding RisCoin-ID provided in the ID-Log and returned the RisCoin-ID with information about a breakthrough infection including the date of the PCR test and the sequencing data to the data manager, where the participant could only be identified by his RisCoin-ID. Unfortunately, the collaboration with the sequencing laboratory did not work properly, resulting in only 9 breakthrough infections identified through this pathway, which had not been reported by the participants via study app or in the follow up questionnaires.
2. Loss of personalized Contact-ID by participants: Participants reported to study team that they had lost their Contact-ID and all study documents and access to their personalized study app, in which the Contact-ID had been stored. If the participant wanted to continue their study participation, they were asked to complete and sign again the ICF including the personal identifiers. A new RisCoin-ID was assigned to this participant, and an ID-Log was generated, which was destroyed after transfer to the Trusted Third party. The study management informed the Trusted Third Party about a person with multiple RisCoin-IDs. The Trusted Third Party matched identifiers participant's (surname, first name, sex, and address) in the electronic ID log and informed the data manager about the duplicate RisCoin-IDs. The data from the two RisCoin-ID files were merged in CentraXX so that the participant could continue participating in the study, access their individual results, and report their vaccination and infection. By July 2022, 28 participants had used this option to re-enter the study as described above after they had irreversibly lost their contact-ID.

After all individual serological results from recruitment and first follow-up had been communicated to the participants, they were informed in May 2022 via study app and intranet that from July 2022 onward, re-identification by the Trusted Third Party would no longer be possible in case of lost Contact-ID. After irreversible anonymization from July 2022 onward, all participants who had lost their Contact-ID were excluded from serological follow-up as it was no longer possible to match their data in RisCoin database.
