# Supplementary material for: A prospective longitudinal cohort study on risk factors for COVID-19 vaccination failure (RisCoin): methods, procedures and characterization of the cohort
Source: Clin Exp Med. 2023 Sep 2;23(8):4901–17. doi: 10.1007/s10238-023-01170-6 (PMC10725370; doi:10.1007/s10238-023-01170-6)
Supplement: Supplementary file 4 — Supplementary file4 (DOCX 67 kb) [file 10238_2023_1170_MOESM4_ESM.docx]

****
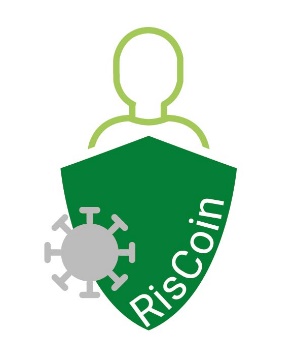
RisCoin-Project Coordination****

PD Dr. Kristina Adorjan

Prof. Dr. Oliver Keppler

Prof. Veit Hornung

Prof. Dr. Sybille Koletzko

Prof. Dr. Matthias Klein

Email: [RisCoin@med.uni-muenchen.de](mailto:RisCoin@med.uni-muenchen.de)

**WELCOME LETTER OF RISCOIN-STUDIE**

Dear study participant,

we are very pleased that you have decided to participate in the RisCoin study! With this, you contribute significantly to better the evidence and control of the COVID-19 pandemic.

**With your personal Contact-ID**, you can access to your individual results or contact the study team in the pseudonymized form.

[123456]

[BARCODE]

Please keep your Contact-ID carefully.

Without this ID, access to your study results is not possible.

Please find below the **link** to the RisCoin initial questionnaire. The personalized link is not transferable to other participants. Answering this questionnaire will take **about 15-20 minutes**. You will be asked about your COVID-19 vaccinations, current intake of medications, vitamins, and supplements. Please have this information ready. You can interrupt at any time and continue later. Your answers will be automatically saved.

**With our team’s assistance, you can active your CentraXX APP by scanning the QR code below.**


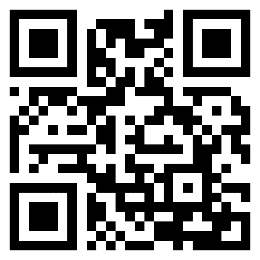


**Please enter your personal link to access the RisCoin initial questionnaire in internet browser**

**https://data.castoredc.com/survey/xxxxx**

**Alternatively, you can also scan the QR Code**


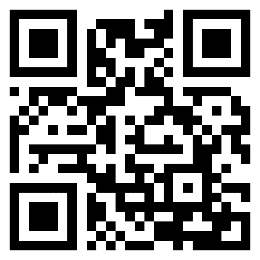


If you have any questions or need assistance, please feel free to reach out to our team under the email [RisCoin@med.uni-muenchen.de](mailto:RisCoin@med.uni-muenchen.de).

Thank you very much for your participation.

Your RisCoin study team

#

# Supplementary information 4: Welcome letter for RisCoin participants

After giving written signed consent, the participants received the individualized **welcome letter** and were provided with the following information (a-c).

1. **Contact-ID**, which replaces their identity and should be saved carefully, because without Contact-ID no communication, including delivery of serological results, is possible.
2. **QR Code to enter the personalized questionnaire** via Castor EDC. The QR code could be scanned, and a personalized internet link was given as an alternative. These two options allowed the participants to answer the questionnaire on a personal computer, tablet, or smartphone.
3. **QR Code to activate the individualized study app**, after downloading to the smartphones via the App Store for iOS or Google Play for Android, respectively. For the few participants without a smartphone or those who did not want to download the study app, the individual entry data via intranet were provided.
